# The molecular mechanism of the cholesterol‐lowering effect of dill and kale: The influence of the food matrix components

**DOI:** 10.1002/elps.201600033

**Published:** 2016-04-24

**Authors:** Francesca Danesi, Marco Govoni, Luigi Filippo D'Antuono, Alessandra Bordoni

**Affiliations:** ^1^ Department of Agri‐Food Science and Technology (DISTAL)University of BolognaCesenaFCItaly; ^2^ BioEngLab, Health Science and Technology – Interdepartmental Center for Industrial Research (HST‐CIRI)University of BolognaOzzano dell'Emilia BOItaly

**Keywords:** Cholesterol, Dill, HepG2, Kale, Quercetin glycosides

## Abstract

Foods are complex matrices containing many different compounds, all of which contribute to the overall effect of the food itself, although they have different mechanisms of action. While evaluating the effect of bioactive compounds, it is important to consider that the use of a single compound can hide the effects of the other molecules that can act synergistically or antagonistically in the same food. The aim of the present study was to evaluate the influence of food matrix components by comparing two edible plants (dill and kale) with cholesterol‐lowering potential and similar contents of their most representative bioactive, quercetin. The molecular effects of the extracts were evaluated in HepG2 cells by measuring the expression of sterol‐regulatory element‐binding proteins (SREBPs), 3‐hydroxy‐3‐methylglutaryl‐CoA reductase (HMGCR) and low density lipoprotein receptor (LDLR) at the mRNA and protein level. The results reported here show that both extracts reduced the cellular cholesterol level with a similar trend and magnitude. It is conceivable that the slightly different results are due to the diverse composition of minor bioactive compounds, indicating that only by considering food as a whole is it possible to understand the complex relationship between food, nutrition, and health in a foodomics vision.

**Abbreviations**
ABCG: ATP‐binding cassette subfamily G
CHOL: cholesterol plus 25‐hydroxycholesterol
Ctrl: control
Ctrl+EtOH: control with ethanol
CVD: cardiovascular disease
d.w.: dry weight
ECACC: European Collection of Authenticated Cell Cultures
HMGCR: 3‐hydroxy‐3‐methylglutaryl‐CoA reductase
IDOL: inducible degrader of LDL receptor
INSIG: insulin induced gene
LDL: low density lipoprotein
LDLR: low density lipoprotein receptor
LXR: liver X receptor
MevaST: mevastatin
mRNA: messenger RNA
MYLIP: myosin regulatory light chain interacting protein
n.s.: not significant
NCD: non‐communicable disease
nSREBP: nuclear form of SREBP
QG: quercetin glycosides
RNA: ribonucleic acid
RT: room temperature
SCAP: SREBP cleavage‐activating protein
SREBF: sterol regulatory element binding transcription factor
SREBP: sterol‐regulatory element‐binding protein

## 1 Introduction

Due to changes in dietary and lifestyle patterns, chronic non‐communicable diseases (NCDs) are becoming increasingly significant causes of disability and premature death in both developing and newly developed countries. Nutrition is considered a major modifiable determinant of NCDs because scientific evidence is increasingly supporting the view that alterations in diet have strong effects, both positive and negative, on health throughout a person's life. Recently, the American Heart Association has indicated the choice of a healthy eating plan as a leading action for cardiovascular disease (CVD) prevention in all age groups 1. Hypercholesterolemia is one of the leading causes of CVD, and it has been suggested that each 1 mmol/L (40 mg/dL) reduction in the levels of low density lipoprotein (LDL) cholesterol decreases the risk of vascular mortality by approximately one‐fifth 2. Consequently, the reduction of elevated LDL cholesterol levels is a significant public health goal. Drugs of the statin class are widely used to lower the plasma LDL cholesterol levels in primary and secondary prevention of CVD 3, but a dietary strategy is desirable due to adverse effects of these drugs. In addition to the maintenance of a normal body weight, the choice of food containing low amounts of saturated fatty acids, trans fatty acids and sugars, and high amounts of polyunsaturated fatty acids and fibres, the consumption of some edible plant species has been considered to have a cholesterol‐lowering potential because they contain bioactive compounds that could act as hypocholesterolemic agents 4. Among them, quercetin, one of the major flavonoids found in fruits and vegetables, has attracted much attention. To date, the effects and mechanism(s) of action of quercetin and other bioactive compounds on the cholesterol plasma level have not been unequivocally shown, and their use still has the potential for health benefits. One limitation to our understanding of bioactive compounds is the indiscriminate use of discrete molecules or the extract of foods rich in bioactive components in the experimental design, which assumes a priori that the other components of the food matrix in which the active molecule is embedded have no influence on the overall effect.

*Anethum graveolens* L. (dill), an annual herb of the *Apiaceae* family, has been reported to lower the blood cholesterol levels in hypercholesterolemic animals 5, 6, 7, 8, 9 and humans 10, 11. *Brassica oleracea* ssp. *acephala* (kale) can favourably affect the serum lipid profiles in hypercholesterolemic 12 and hypertensive patients 13. The aim of the present study was to evaluate the influence of the food matrix components by comparing two edible plants – dill and kale – with cholesterol‐lowering potential and similar contents of their most representative bioactive compound, quercetin glycosides (QG). HepG2 cells were used as a model system, and cholesterol and mevastatin treatments were used as positive and negative *stimuli*, respectively.

The molecular effects of the two extracts were investigated at the mRNA and protein level, focusing on sterol regulatory element binding proteins (SREBPs), hydroxy‐3‐methylglutaryl‐CoA reductase (HMGCR), and low density lipoprotein receptor (LDLR). SREBPs are transcription factors whose activation is tightly regulated by the cellular sterol concentration, and are considered master regulators of lipid homeostasis 14. HMGCR and LDLR have a central role in cholesterol synthesis and trafficking, and their transcriptional regulation is mediated by SREBP.

## 2 Materials and methods

### 2.1 Materials

All chemicals, reagents, and solvents were purchased from Sigma‐Aldrich Co. (St. Louis, MO, USA), unless otherwise stated. All aqueous solutions were prepared using ultrapure water (Milli‐Q; Millipore, Bedford, CT, USA). Stock solutions of cholesterol (2 mg/mL), 25‐hydroxycholesterol (0.2 mg/mL) and mevastatin (0.002 mg/mL) were prepared in ethanol, aliquoted and stored at –20°C until use.

### 2.2 Plant materials and extracts

All plant materials were sourced from areas of the Black Sea region. Dried plant materials were processed as previously described 15, and the crude methanolic extracts were analysed for their polyphenolic composition by HPLC‐DAD‐MS. The details of the analytical conditions are reported in Hollands et al. 15, and the bioactive compound composition of the plant extracts is reported in Table 1.

**Table 1:** Concentrations of the main bioactive compounds in the plant extracts used to supplement the cells (adapted from 15)

Dill extract	Kale extract
Phenolic acids:	Phenolic acids:
Chlorogenic 21.43 mg/g extract	Chlorogenic 3.77 mg/g extract
Feruloyl quinate 1.63 mg/g extract	
Flavonols:	Flavonols:
Quercetin glycosides 32.07 mg/g extract	Quercetin glycosides 31.81 mg/g extract
	Gentiobioses: 4.81 mg/g extract
	Glucosinolates: 7.66 μmol/g d.w.

The crude methanolic extracts were dissolved in ultrapure water at a concentration of 10 mg/mL; clear solutions were prepared by gentle heating (at 40°C) and stirring. The stock solutions were filtered through sterile 0.22 μm filters, aliquoted and stored at −20°C until further use.

### 2.3 Cell culture

HepG2 cells were obtained from the European Collection of Authenticated Cell Cultures (ECACC; Salisbury, UK), and the cells were grown in DMEM (Lonza, Basel, Switzerland) supplemented with 10% foetal bovine serum, 100 U/mL of penicillin, and 100 μg/mL of streptomycin. Cells were seeded at a density of 1.0×10^6^ cells per well in 6−well plates (for cholesterol quantification and RNA isolation) or at 3.0×10^6^ cells in 10 cm dishes (for protein extraction). Twenty‐four hours after seeding, the media was removed, and the cells were randomly divided into four groups and refed with serum‐free media that was supplemented with dill extract (DILL, 100 μg/mL), kale extract (KALE, 100 μg/mL), cholesterol plus 25‐hydroxycholesterol (CHOL, 10 μg/mL + 1 μg/mL), or mevastatin (MevaST, 0.05 μM).

The selected 100 μg/mL dose of the extracts (corresponding to ≈ 6 μM QG) is similar to the peak plasma concentrations in humans after the consumption of quercetin‐fortified foods or supplements 16, 17. Furthermore, in preliminary experiments using scalar concentrations (50 μg/mL − 1 mg/mL) of the extracts for 24 h, the 100 μg/mL dose did not cause any cytotoxic effects (data not shown). The combination of cholesterol and 25‐hydroxycholesterol was utilized to induce cholesterol over‐loading 18, 19, 20, while the mevastatin treatment was applied to cause cholesterol‐lowering effects 21. Because the kale and dill extracts were dissolved in water and cholesterol plus 25‐hydroxycholesterol and mevastatin were dissolved in ethanol, two control conditions were considered: the first one received water (Ctrl) and the second received ethanol (≤ 1% v/v; Ctrl+EtOH). In all experiments, the cells were incubated in a serum‐free medium to prevent the serum lipoproteins from interfering with intracellular gene expression 22. Twenty‐four hours after supplementation, the cells were washed twice with ice‐cold 0.9% NaCl, and the subsequent analyses were performed.

### 2.4 Evaluation of the cellular cholesterol content

Total cholesterol was measured using the Amplex Red Cholesterol Assay Kit (Life Technologies Inc.; Camarillo, CA, US) according to the manufacturer's instructions. Briefly, the cells were harvested in 1 mL of ice‐cold PBS. Cell pellets were collected by centrifugation at 250×*g* for 5 min and then dissolved in 2.5 mL of hexane/isopropanol (3:2) for 15 min to extract the lipids. One mL of chloroform containing 2% Triton X‐100 was added to enhance the extraction 23, 24. After centrifugation at 1,500×*g* for 5 min, the organic layer was separated and dried under nitrogen. The lipids were resuspended in 500 μL of 1X reaction buffer (0.1 M of potassium phosphate buffer, pH 7.4, with 0.05 M of NaCl, 5 mM of cholic acid, and 0.1% Triton X‐100) and stored at –20°C until use.

The cholesterol detection assay was conducted in a 96−well microplate using a 100‐μL reaction volume per well. Fifty microliters per well of a 300 μM Amplex Red Reagent working solution containing 2 U/mL of horseradish peroxidase, 2 U/mL of cholesterol oxidase, and 0.2 U/mL of cholesterol esterase were added to 50 μL of an undiluted sample or standard. The assay was incubated with esterase to quantify the total cholesterol levels. After a 30‐min incubation in the dark at 37°C, the fluorescence of each sample was measured using a Tecan Infinite F200 microplate reader (Tecan; Salzburg, Austria) that was equipped with a filter set for excitation and emission at 535 ± 25 and 595 ± 25 nm. The values obtained from a cholesterol standard curve (0–8 μg/mL) were normalized to the protein content measured using the Bio‐Rad dye‐binding protein assay (Bio‐Rad Laboratories; Hercules, CA, US), and results were expressed as a percentage relative to the corresponding control cells.

### 2.5 Quantitative real‐time PCR (qPCR)

The cells were lysed directly in the culture dish, and the total RNA was isolated using the RNeasy kit (Qiagen; Hamburg, Germany) according to manufacturer's protocol. The quantity and quality of the RNA were assessed using the NanoDrop ND‐2000 spectrophotometer (Thermo Fisher Scientific; Wilmington, DE, US). The samples were frozen at −80°C until use.

Reverse‐transcription and qPCR were performed as previously described 25. The reference genes (β‐actin, glyceraldehyde‐3‐phosphate dehydrogenase, hydroxymethylbilane synthase, and subunit A of the succinate dehydrogenase complex) were selected based on previously published literature 26 and designed based on published sequences 25. Specific primers for the target genes were designed using the publicly available web‐based Primer3 program (http://frodo.wi.mit.edu/primer3). Each primer set was checked for specificity using the basic local alignment sequence tool (http://www.ncbi.nlm.nih.gov/tools/primer‐blast). The primer set for sterol regulatory element binding transcription factor 1 (*SREBF1*) was designed to overlap a site common to both splice variants *SREBF1a* and *1c*. See Table 2 for the primer sequences of the target genes. All oligonucleotides were synthesized by Integrated DNA Technologies (Leuven, Belgium). The specificity of the qPCR products was confirmed by a melting curve analysis and electrophoresis on a gel, as previously reported 25.

**Table 2:** Primer sequences of the target genes analysed by qPCR

Gene name	GenBank accession number	Primer sequencea)	Amplicon size
*HMGCR*, 3‐hydroxy‐3‐methylglutaryl‐CoA reductase	NG_011449.1	F: TGTCAGGGGTACGTCAGCTT	102 bp
		R: AGGACACACAAGCTGGGAAG	
*LDLR*, low density lipoprotein receptor	NG_009060.1	F: CCAGCCAAGAGGAGTGAAC	117 bp
		R: CAGGCGCAGGTAAACTTGG	
*SREBF1*, sterol regulatory element binding transcription factor 1	NG_029029.1	F: TCCACAAAAGCAAATCTCTGAA	96 bp
		R: CCTCCACCTCAGTCTTCACG	
*SREBF2*, sterol regulatory element binding transcription factor 2	NC_000022.11	F: AAAGGCGGACAACCCATAAT	111 bp
		R: ACTTGTGCATCTTGGCGTCT	

a) F: forward primer; R: reverse primer.

The data were analysed using the DataAssist Software version 3.01 (Applied Biosystems, Foster City, CA, US) and expressed as the mean fold change in relative expression compared with the respective control cells (Ctrl or Ctrl+EtOH), which were normalized to one.

### 2.6 Western blot analysis

The cells were harvested in 3 mL of ice‐cold PBS. The cell pellets were collected by centrifugation at 250×*g* for 5 min at 4°C and then lysed in a Nonidet‐P40 buffer (150 mM of sodium chloride, 1% Triton X‐100, and 1% protease inhibitor cocktail in 50 mM of Tris pH 8.0). The cell lysates were passed through a 30 gauge needle, sonicated at 38 kHz for 5 min, and centrifuged at 14,000 × *g* for 15 min at 4°C. The concentration of the proteins in the supernatant was determined according to the Bradford method 27.

The proteins were diluted 1:1 with a loading buffer and then denatured by boiling, as previously described 28. Aliquots containing 50 μg of total protein were loaded onto a 10% SDS‐polyacrylamide gel and blotted onto a nitrocellulose membrane. After being blocked with non‐fat dry milk for 60 min at room temperature (RT), the membrane was probed with specific anti‐HMGCR (1:500), anti‐LDLR (1:500), anti‐SREBP1 (1:200), and anti‐SREBP2 (1:200) primary antibodies (Abcam, Cambridge, UK) overnight at 4°C. After additional washes, the membrane was incubated with the horseradish peroxidase‐conjugated secondary antibody (Abcam) for 60 min at RT. Then, the immunoreactive bands were visualized with an enhanced chemiluminescence kit (GE Healthcare, Buckinghamshire, UK). A mouse monoclonal antibody raised against β‐actin (Sigma‐Aldrich Co.) was used as a protein‐loading control. The protein expression levels were obtained by densitometric quantification with a GS‐800 Calibrated Densitometer and Quantity‐One software (Bio‐Rad Laboratories). The protein levels are expressed as the fold change compared with the respective control (Ctrl or Ctrl+EtOH) upon normalization for β‐actin.

### 2.7 Statistical analysis

The data are reported as the means ± SD of four to six biological replicates from two independent cell cultures. The differences among the treatments were evaluated by one‐way ANOVA followed by Dunnett's test and compared with the respective control cells (Ctrl or Ctrl+EtOH), with *p*<0.05 considered significant.

## 3 Results and discussion

The aim of the present study was to compare the effectiveness as hypocholesterolemic agents of two extracts having a similar total phenolic and QG content, although characterized by the presence of different peculiar components (Table 1). As previously reported by Hollands and colleagues 15, the main bioactive compounds in the dill extract were QG and chlorogenic acid, while the kale extract mainly contained QG and glucosinolates, and a small amount of chlorogenic acid.

**Figure 1:**
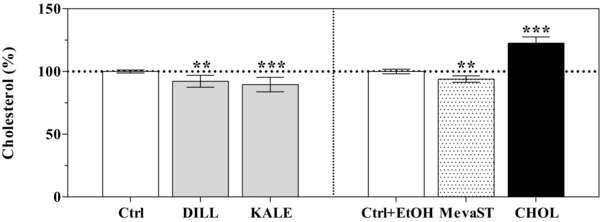
Intracellular cholesterol levels of the unsupplemented (Ctrl, Ctrl+EtOH), supplemented (DILL and KALE) and treated (CHOL and MevaST) cells. The data are expressed as the percentage of the value obtained in the respective control cells (Ctrl or Ctrl+EtOH), which are set to 100% and are the means ± SD of six samples in each group. The statistical analysis was performed using one‐way ANOVA (*p*<0.001) followed by Dunnett's test (** *p*<0.01, *** *p*<0.001 vs. the respective control). No differences were detected between the Ctrl and Ctrl+EtOH cells.

Compared with the corresponding controls, the cells supplemented with dill and kale showed a significant reduction in their cholesterol levels (–7.8 and –10.4%, respectively), which was similar to MevaST (–6.0%); on the contrary, treatment with CHOL significantly increased the cholesterol concentration (+22.6%) (Fig. 1).

**Figure 2:**
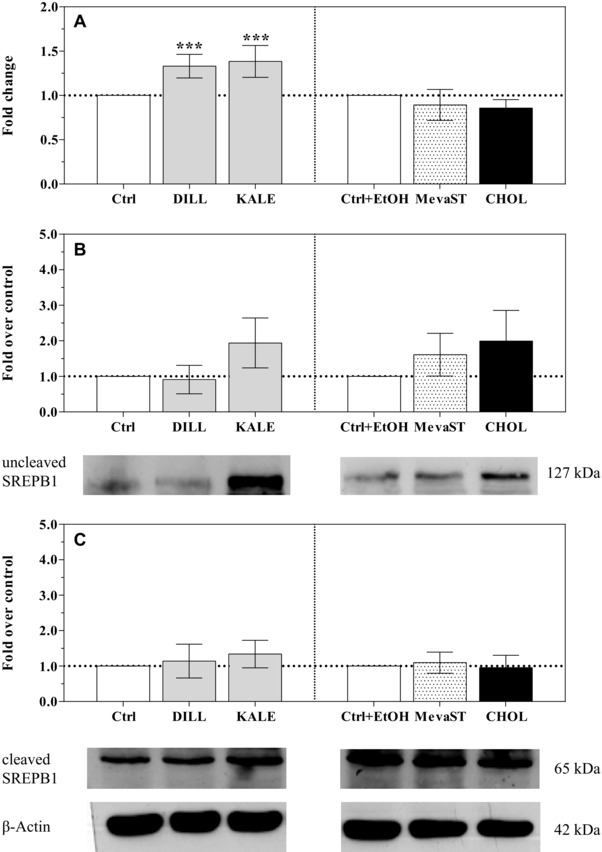
*SREBF1* mRNA expression (A) and uncleaved and cleaved SREBP1 protein levels (B and C, respectively) in the unsupplemented (Ctrl, Ctrl+EtOH), supplemented (DILL and KALE) and treated (CHOL and MevaST) cells. The data in panel A are expressed as the mean fold change relative to the respective control cells (Ctrl or Ctrl+EtOH), which are normalized to one and are the means ± SD of six samples in each group. The data in panel B are expressed as a SREBP1/β‐actin ratio, compared with the respective control cells (Ctrl or Ctrl+EtOH), which are normalized to one and are the means ± SD of four samples in each group. The statistical analysis was performed using one‐way ANOVA (panel A *p*<0.001; panel B and C n.s.) followed by Dunnett's test (*** *p*<0.001 vs. the respective control). No differences were detected between the Ctrl and Ctrl+EtOH cells.

**Figure 3:**
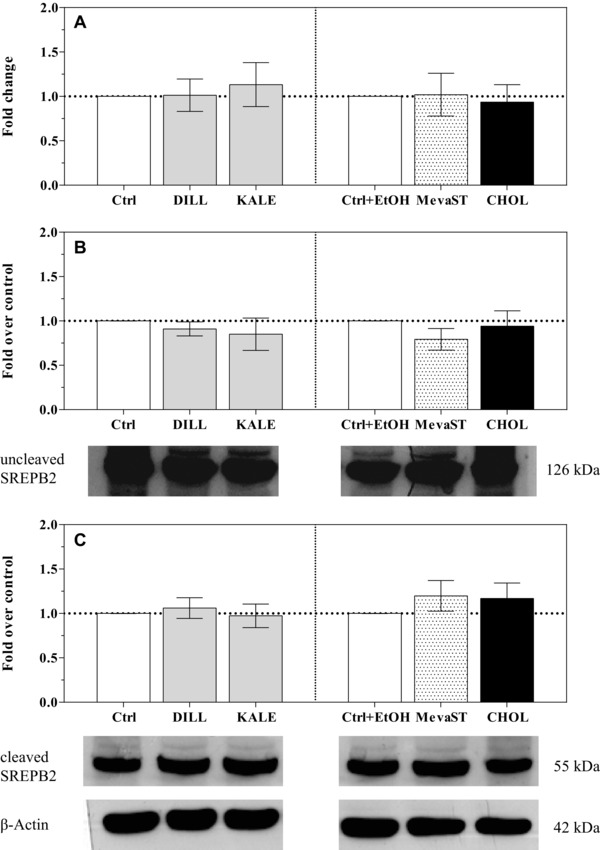
*SREBF2* mRNA expression (A) and uncleaved and cleaved SREBP2 protein levels (B and C, respectively) in the unsupplemented (Ctrl, Ctrl+EtOH), supplemented (DILL and KALE) and treated (CHOL and MevaST) cells. The data in panel A are expressed as the mean fold change relative to the respective control cells (Ctrl or Ctrl+EtOH), which are normalized to one and are the means ± SD of six samples in each group. The data in panel B are expressed as a SREBP2/β‐actin ratio compared with the respective control cells (Ctrl or Ctrl+EtOH), which are normalized to one and are the means ± SD of four samples in each group. The statistical analysis was performed using one‐way ANOVA (panel A, B, and C n.s.) followed by Dunnett's test (n.s. vs. the respective control). No differences were detected between the Ctrl and Ctrl+EtOH cells.

Based on the observed modifications in the intracellular cholesterol levels, we expected that the SREBPs would be modulated in our experimental conditions. SREBPs are transcription factors that are considered master regulators of intracellular cholesterol homeostasis. When the cellular cholesterol level is low, a SREBP cleavage‐activating protein (SCAP) escorts the SREBPs from the endoplasmic reticulum to the Golgi, where the SREBPs are cleaved by proteases into the mature form (nSREBP) that translocates to the nucleus and binds to the sterol regulatory element, thus triggering the transcription of genes required for cholesterol synthesis and trafficking 14. On the contrary, when the cholesterol level exceeds the cellular demand, the SCAP‐SREBP complex is sequestered in the endoplasmic reticulum by the insulin‐induced gene product known as insulin‐induced gene 1 (INSIG1). Although the CHOL and MevaST treatments caused a huge modification of the cholesterol levels, the modulation of the expression or the activation of SREBP1 and two have not been triggered (Figs. 2 and 3, respectively). The lack of a modulation may be ascribed to a transient effect on SREBP transcription and translation, not detectable after 24 h exposure, or to an SREBP degradation by proteasome 29.

The expression of *SREBF1* was only upregulated in the dill‐ and kale‐supplemented cells (Fig. 2A). Notwithstanding the increased *SREBF1* transcription, there was no detectable modification in the level of the encoded protein (Fig. 2B and C). Although it has been reported that supplementation with quercetin as a discrete molecule decreases SREBP1c expression both at the protein and mRNA level in primary rat hepatocytes 30 and in mouse adipocytes 31, our results are consistent with the reported increase in liver X receptor (LXR) α expression and activation by quercetin 32, 33. LXR activation potently upregulates *SREBF1c* expression 34. Phytochemicals other than quercetin in the dill and kale extracts could have synergistically contributed to LXR activation by sustaining its expression to allow it to upregulate *SREBF1c* expression.

**Figure 4:**
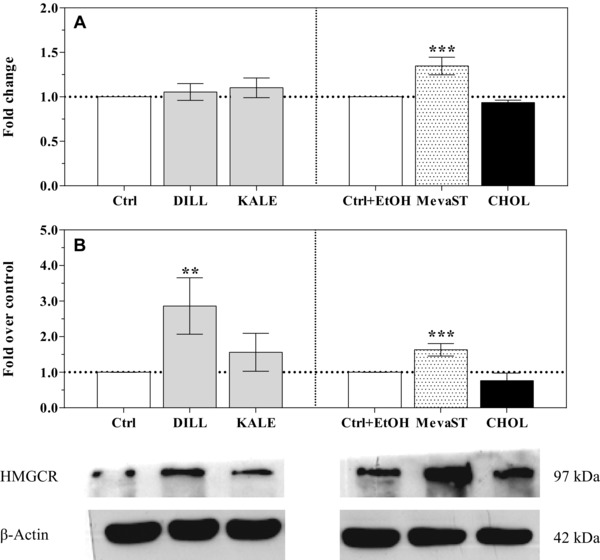
*HMGCR* mRNA expression (A) and HMGCR protein levels (B) in the unsupplemented (Ctrl, Ctrl+EtOH), supplemented (DILL and KALE) and treated (CHOL and MevaST) cells. The data in panel A are expressed as the mean fold change relative to the respective control cells (Ctrl or Ctrl+EtOH), which are normalized to one and are the means ± SD of six samples in each group. The data in panel B are expressed as an HMGCR/β‐actin ratio compared with the respective control cells (Ctrl or Ctrl+EtOH), which are normalized to one and are the means ± SD of four samples in each group. The statistical analysis was performed using one‐way ANOVA (panel A and B *p*<0.001) followed by Dunnett's test (** *p*<0.01, *** *p*<0.001 vs. the respective control). Significant differences were detected when comparing Ctrl and Ctrl+EtOH (*p*<0.05).

**Figure 5:**
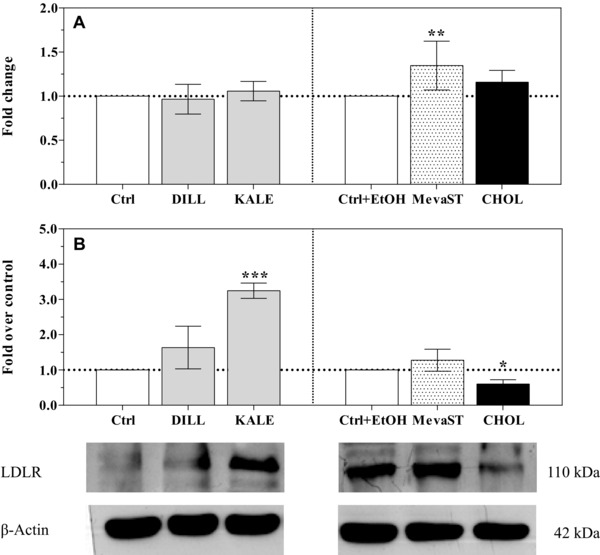
*LDLR* mRNA expression (A) and LDLR protein levels (B) in the unsupplemented (Ctrl, Ctrl+EtOH), supplemented (DILL and KALE) and treated (CHOL and MevaST) cells. The data in panel A are expressed as the mean fold change relative to the respective control cells (Ctrl or Ctrl+EtOH), which are normalized to one and are the means ± SD of six samples in each group. The data in panel B are expressed as an LDLR/β‐actin ratio compared with respective control cells (Ctrl or Ctrl+EtOH), which are normalized to one and are the means ± SD of four samples in each group. The statistical analysis was performed using one‐way ANOVA (panel A *p*<0.01; panel B *p*<0.001) followed by Dunnett's test (* *p*<0.05, ** *p*<0.01, *** *p*<0.001 vs. the respective control). Significant differences were detected between the Ctrl and Ctrl+EtOH cells (*p*<0.01).

With the exception of the mevastatin‐treated cells, the transcription of the *HMGCR* and *LDLR* genes in all supplemented cells was similar to the controls (Figs. 4A and 5A, respectively). The reported results on the effects of mevastatin treatment on the expression of the *HMGCR* and *LDLR* genes are in agreement with the current literature 35, 36, 37.

At the protein level, HMGCR expression was significantly increased by dill supplementation, and a similar trend was observed in the kale‐supplemented cells (Fig. 4B). Similarly, the LDLR protein level appeared to be significantly increased in the kale‐supplemented cells, and the same trend was present but not significant in the dill‐supplemented cells (Fig. 5B). It is understood that modifications in gene expression and protein abundance may not correlate; the possible biological reasons for this poor correlation include the possibility that proteins have different half‐lives as well as different post‐transcriptional and post‐translational modifications.

The increased HMGCR and LDLR protein expression was not accompanied by the upregulation of the expression of the corresponding gene, which could be due to a reduction in protein hydrolysis via the regulation of the ubiquitin–proteasome system by the dill and kale bioactive compounds. It has been reported that LDLR expression is also regulated by protein ubiquitination via the ubiquitin ligase MYLIP/IDOL 38, which is induced by LXR 39. As reported above, quercetin increases the expression of and activates LXRα 32, 33.

Independent of the underlying mechanisms, the expression of the key enzyme in cholesterol de novo biosynthesis and of the receptor involved in cholesterol uptake was upregulated by the dill and kale extracts in a similar but not identical way. It is conceivable that quercetin is primarily responsible for these effects, but other bioactive compounds in the extracts tune its action.

The increase HMGCR and LDLR protein levels in the dill‐ and kale‐supplemented cells appear to contrast the observed reduced intracellular cholesterol concentrations. In humans, hepatocytes are important for de novo cholesterol biosynthesis, which accounts for approximately 10% of the biosynthesis, as well as for the maintenance of normal blood cholesterol levels via the clearance of plasma LDL cholesterol through the LDLR 40, and hepatic cells are furthermore important for promoting cholesterol biliary excretion. The reverse cholesterol transporters ATP‐binding cassette subfamily G members (ABCG5/ABCG8) are glycoproteins whose expression is modulated by LXR 41. Using artichoke leaf extracts, Gebhardt 42 showed that chlorogenic acid stimulates biliary secretion, although to a lesser extent than lutein. Furthermore, in a very recent paper, Hao et al. 43 showed that the efflux of total cholesterol in HepG2 cells is increased by chlorogenic acid. Accordingly, in our study, an enhanced cholesterol efflux mediated by chlorogenic acid could have prevented the increase in the cholesterol cellular concentration due to the overexpression of HMGR and LDLR induced by quercetin.

## 4 Concluding remarks

Foods are complex matrices containing many different bioactive compounds, all of which contribute to the overall effect of the food itself even though they have different mechanisms of action. Foodomics is the discipline that studies food and nutrition through the application of omics technologies 44, and it is greatly contributing to the improvement of food science and has the goal of improving human nutrition. A major limitation and current barrier to progress in food science and nutrition is that it is seldom considered that the molecular effect of a bioactive food component is predictable and reproducible only in theory because in practice, it can be modified by the food matrix effect, which is the influence of other food components 45. This study represents a first step in unravelling the complex food matrix effect.

When evaluating the effects of bioactive compounds, it is important to consider that the use of single compounds can hide the effects of other molecules that are present in the same food. Therefore, the use of extracts instead of pure molecules is not a limitation because the activity of a whole food needs to be deciphered at the molecular level. To the authors’ knowledge, this is the first study to compare the molecular effects of different foods that share the same molecule as the major bioactive component but significantly differ in the composition of the other bioactive compounds. Although we obtained results with a similar magnitude and trend in the dill‐ and kale‐supplemented cells, the slight modifications by the different compositions of the minor bioactive compounds were clearly shown, indicating that only by considering the food as a whole is it possible to understand the complex relationship between food, nutrition and health in a foodomics vision.

*This study was funded by the European project BaSeFood “Sustainable exploitation of bioactive components from the Black Sea Area traditional foods” (EC Contract no: FP7‐KBBE‐227118) and by the Italian Ministry of Education, University and Research (MIUR; RFO fund to A.B.). The authors thank Dr. Nadiya Boyko, Dr. Mariia Mudryk (Uzhhorod National University, Ukraine), Dr. Bike Kocaoglu and Dr. Osman Hayran (Yeditepe University, Turkey) for providing the dill and kale samples, respectively. The authors thank Dr. Marija Glibetić (University of Belgrade, Serbia) and Dr. Paul A. Kroon (Institute of Food Research, UK) for preparing the dill and kale extracts, respectively. The authors also wish to thank Dr. Dario de Biase (University of Bologna, Italy) for providing excellent technical assistance with the real‐time PCR assay*.

*All authors state that they have no conflicts of interest to declare that are related to this study*.
